# Computational and experimental study of thermal behavior in the oral cavity for biosensing applications

**DOI:** 10.1016/j.rineng.2025.105284

**Published:** 2025-06

**Authors:** Yuanzhe Zhao, Jeroen H.M. Bergmann

**Affiliations:** a Department of Engineering Science, University of Oxford, Oxford, UK; b Department of Technology & Innovation, University of Southern Denmark, Odense, Denmark

**Keywords:** Core temperature, Body temperature, Oral temperature, Heat transfer, Simulation, Temperature distribution

## Abstract

This study investigates transient heat transfer in the oral cavity under varying conditions using a finite element model combined with corresponding experiments. By modeling and measuring oral temperature during cold liquid intake, temperature recovery, and prolonged cold air inhalation, this study examines both temperature distribution across different oral regions and transient thermal dynamics. Results reveal that anterior teeth exhibit greater thermal sensitivity and slower temperature recovery following cold exposure, while posterior regions maintain higher and more stable temperatures. Additionally, buccal surfaces consistently show higher and more stable temperatures than lingual surfaces, underscoring the importance of measurement location for accuracy. The simulation demonstrated moderate agreement with experimental data, achieving a mean absolute error (MAE) of 3.39 °C (13.46%) for upper facial positions and 2.91 °C (10.35%) for lower facial positions during cold liquid intake scenarios. Furthermore, a strong correlation was observed between core body temperature and regional oral temperatures during prolonged cold air inhalation. While the total duration of mouth breathing significantly affects oral temperature, variations in respiratory cycle frequency showed minimal impact on thermal response. These findings provide important guidance for the development of non-invasive core temperature estimation methods based on oral biosensors. In particular, they offer insights for optimizing sensor placement in thermally stable regions and designing data-driven calibration algorithms capable of compensating for transient environmental disturbances, thereby enhancing the accuracy and reliability of wearable biosensing systems in dynamic conditions.

## Highlights

• A computational model simulates oral temperature under various conditions.
• Front teeth show slower temperature recovery and greater fluctuation.
• Buccal surfaces maintain higher, more stable temperatures than lingual surfaces.
• Oral cavity temperature returns to near core temperature within 15 minutes after cold exposure.
• Respiratory rate has minimal impact on oral temperature during breathing.

## 1 Introduction

Body temperature, a fundamental vital sign, serves as a crucial indicator of human health [1]. Alterations in body temperature often signal the onset of diseases, particularly those affecting the internal organs. These temperature changes are a response to various stimuli, such as the invasion of pathogenic bacteria. Such invasions trigger a neuroendocrine response, potentially leading to a significant stress response and subsequent changes in body temperature [2], [3]. The precision of body temperature measurements is thus integral to the accurate diagnosis and treatment of illnesses. Inaccurate measurements can not only hinder effective treatment but also pose serious risks to patient safety [4]. Beyond its role in medical diagnostics, precise body temperature monitoring is critical in sports and physical activities. During intense or prolonged exercise, body temperature naturally rises. This increase is particularly concerning in hot and humid conditions, common in many major sporting events [5], [6]. High environmental temperatures can severely strain the body's thermoregulatory and cardiovascular systems, as well as impact central nervous system function, metabolic processes, muscle function, and fluid balance. These effects heighten the risks of exercise-induced complications such as muscle cramps, heat syncope, heat exhaustion, and even heatstroke [7], [8]. Consequently, accurate body temperature monitoring is essential not only for optimal performance but also for athlete safety and the prevention of long-term health issues [9].

Traditional accurate core body temperature measurement methods usually include rectal, esophageal, and pulmonary artery temperature measurement [10], [11]. Although these methods can provide relatively accurate readings, their invasiveness and operational complexity limit their widespread application in daily life [12]. Therefore, finding a non-invasive, convenient and accurate method for measuring core body temperature has become a research direction that has attracted much attention. Common measurement sites, including the axilla, oral cavity, and tympanic membrane or skin surfaces, offer greater convenience but often show poor agreement with direct core temperature measurements, especially under conditions involving physical activity or environmental stress [13], [14]. To address this discrepancy, recent research has increasingly focused on leveraging wearable biosensors combined with data-driven algorithms to estimate core body temperature from these accessible sites such as [15], [16], [17].

In recent years, the oral cavity has emerged as a promising platform for wearable biosensors due to its unique anatomical and physiological characteristics [18]. Devices such as mouthguard with sensors or dental implants have been developed not only for temperature monitoring but also for tracking biomarkers, hydration levels, pH, and even respiratory patterns [19], [20], [21]. This integration of sensors into intraoral devices offers significant advantages in terms of user comfort, discretion, and the ability to function effectively during physical activity, making them ideal for applications in sports science, healthcare, and remote patient monitoring.

Oral temperature measurement has become a potential solution for core body temperature estimation due to its convenience and low invasiveness. However, the complexity of the oral environment and the problem of susceptibility to interference from external factors have challenged the accuracy of this solution in actual use [22], [23], [24]. In recent years, advances in biosensor technology have led to the development of various intraoral devices aimed at continuous temperature tracking [19], [25], [20], [26], [27]. However, ingesting hot or cold liquids or breathing cold air through the mouth can significantly affect the accuracy of these measuring devices [28], [29]. Due to the heat transfer between the oral tissue and the external fluid, factors such as the fluid temperature and exposure time can cause drastic fluctuations in the local temperature in the mouth, which in turn causes the temperature change to fail to accurately reflect the core body temperature. This temperature interference caused by the external environment limits the measurement accuracy of existing devices in actual scenarios such as ingesting hot or cold fluids or inhaling cold air.

In order to develop the core temperature estimation method from oral temperature, it is particularly important to understand the transient heat transfer process in the oral cavity. By analyzing the dynamic changes in the temperature inside the oral cavity when taking in hot or cold liquids or inhaling cold air, a theoretical basis can be provided for designing more robust methods. This analysis not only helps to optimize the layout of sensors, but also provides strong support for improving the rapid response capability and anti-interference ability of sensors in the future.

Existing experimental studies have explored the heat transfer phenomenon in the oral cavity to varying degrees. Some studies have analyzed the temperature changes of teeth and tongue after taking in cold or hot liquids through experimental methods, revealing the thermal response characteristics of oral tissues under different temperature environments [28], [29], [30], [31]. These studies provide preliminary data support for understanding the thermal behavior in the oral cavity. However, the research on the transient temperature response of the oral cavity when breathing cold air, especially in terms of sensor layout and core body temperature estimation, is still relatively limited. In addition, most existing studies focus on the measurement of steady-state temperature and lack in-depth analysis of transient temperature changes. In actual use, the rapid change of temperature in the oral cavity is likely one of the key factors affecting sensor performance.

Finite element modeling (FEM) has been widely applied in biomedical thermal studies to simulate complex heat transfer processes within human tissues. Previous FEM-based research related to oral and airway thermodynamics has primarily focused on specific anatomical structures or steady-state conditions. For instance, several studies have modeled tongue temperature distribution to assist in disease diagnosis, such as detecting oral cancer [32], [33]. Other works have concentrated on simulating heat transfer within the upper respiratory tract to evaluate thermal injury risks during exposure to extreme air temperatures [34], [35]. Additionally, FEM has been employed to analyze heat conduction within dental tissues, supporting the development of dental treatment strategies under controlled thermal environments [36], [37]. However, most of these studies are limited to localized regions or steady-state scenarios, largely due to the complexity involved in modeling transient heat transfer coupled with fluid-structure interactions [38].

To the best of our knowledge, no prior research has comprehensively investigated the transient thermal behavior of the entire oral cavity under dynamic cold exposure, such as fluid intake or mouth breathing. This gap highlights the need for advanced modeling approaches to better understand the unsteady thermal dynamics affecting oral temperature measurements.

The main objective of this study is to investigate the transient heat transfer processes within the oral cavity during cold fluid ingestion and cold air inhalation using finite element analysis (FEM) combined with volunteer experiments. Specifically, we aim to evaluate the temperature responses at different oral locations under dynamic thermal conditions.

Particularly, this study explores the impact of respiratory frequency and exposure duration on oral temperature variations during cold air breathing, providing insights into how these physiological factors influence transient thermal behavior.

The findings of this research will offer a theoretical foundation for optimizing the design of oral temperature monitoring devices, particularly in terms of sensor placement, calibration strategies, and algorithm development. By addressing the challenges posed by external thermal disturbances and physiological variability, this work contributes to enhancing the accuracy and reliability of oral-based core body temperature estimation in real-world scenarios.

## 2 Method

### 2.1 Computational study

This study simulated the heat transfer between fluid flow in the mouth and oral tissue, focusing on the temperature response of oral tissue during the inhalation of cold water or air. The model was built in COMSOL Multiphysics 6.1, and simulated by coupling laminar flow and bio-heat transfer interface.

#### 2.1.1 Geometry structure and material properties

**Fig. 1:**
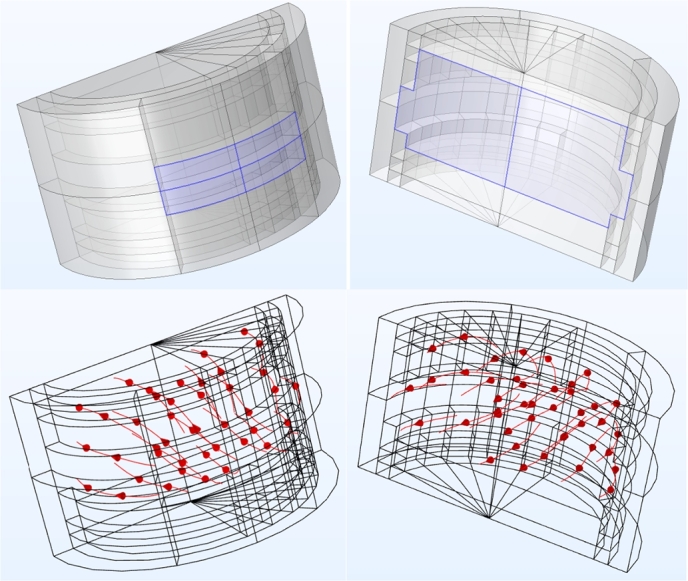
The simplified 3D oral structure model with blue representing the openings and the fluid flow direction.

As depicted in Fig. 1, a simplified 3D oral structure model was established to simulate the transient heat transfer process in the oral cavity and the fluid inlet is represented by the smaller blue area (representing the mouth). In order to reduce the computational complexity and ensure the validity of the simulation results, the model includes key oral anatomical as basic structures. These simple shapes represent the teeth, tongue, and oral mucosa, as well as skin. The fluid flow direction is also shown in Fig. 1.

This geometric model is based on publicly available oral anatomical data and simplifies the internal structure of the oral cavity to ensure the feasibility and rationality of the model calculation [39], [40], [41], [42]. The material properties in the model are set according to experimental data in the literature [43], [44]. The basic parameters are shown in Table 1. In this study, oral tissues were modeled as rigid bodies. However, future studies are expected to incorporate mechanical deformation of oral tissues caused by fluid flow or muscle movement.

**Table 1:** The basic parameters of materials.

Material	Tooth	Tongue	Mucosa/Skin
Density (kg/m^3^)	2200	1040	1100
Thermal conductivity (W/m⋅K)	0.88	0.5	0.45
Specific heat (J/(kg⋅K))	1260	3600	3500
Blood perfusion rate (1/s)	0	0.0005	0.0018
Metabolic heat (W/m^3^)	0	500	1300

Blood density (kg/m^3^)	1000		
Blood heat capacity (J/(kg⋅K))	4000		
Arterial blood temperature (K)	310.15 K (37 °C)		

#### 2.1.2 Governing equations

To take into account the effects of fluid flow, we consider the following three-dimensional incompressible Navier-Stokes equations and laminar flow [45], [46]:

(1) $$\rho_{f}\frac{\partial u}{\partial t}+\rho_{f}(u\cdot \nabla )u=\nabla \cdot \left[-pI+K\right]+\rho_{f}g,$$

(2) $$\rho_{f}(\nabla \cdot u)=0$$

Where *t* is the time, $\rho_{f}$ is the density of the fluid (kg/m^3^), **u** is the velocity field of air (m/s), *p* is the pressure (Pa), **I** is the identity tensor, **K** is the stress tensor (Pa), and **g** denotes the gravity acceleration (m/s^2^).

The Pennes' bioheat transfer model is based on Fourier's law, which assumes that heat propagation occurs at infinite speed [47]:

(3) q(r,t)=−k∇T(r,t)

Where ***q*** is the heat flux vector, *k* is the thermal conductivity, *T* is the temperature, and ***r*** is the position vector.

The transient heat transfer in the oral tissue (tooth, tongue and mucosa/skin) can be described as Pennes' Bioheat Transfer Equation [46], [47]:

(4) $$\rho_{o}C_{p,o}\frac{\partial T}{\partial t}+\nabla \cdot (-k_{o}\nabla T)=\rho_{b}C_{p,b}\omega_{b}(T_{b}-T)+Q_{\text{met}}$$

Where:

• $\rho_{o}$ is the density of the oral tissue (kg/m^3^),
• $C_{p,o}$ is the specific heat capacity of the oral tissue (J/(kg⋅K)),
• $k_{o}$ is the thermal conductivity of the oral tissue (W/(m⋅K)),
• $\rho_{b}$ is the density of the blood (kg/m^3^),
• $C_{p,b}$ is the specific heat capacity of the blood (J/(kg⋅K)),
• $\omega_{b}$ is the perfusion speed of the blood (m/s),
• $Q_{\text{met}}$ is the metabolic heat (W).

This equation takes into account heat conduction in tissues, the effects of blood perfusion convection, and the generation of metabolic heat.

The human body exchanges heat with the environment through the skin surface by radiation, convection, evaporation, etc. In this model, only the cooling/heating effect caused by the flow of fluid in the oral cavity is considered.

#### 2.1.3 Boundary conditions

At all external boundaries of the oral tissue, the temperature was set to a constant value equal to the arterial blood temperature. Similarly, the initial temperature for all domains, both fluid and oral tissue, was assumed to be the arterial blood temperature.

For the fluid domain, the inflow temperature was set to 0 °C (273.15 K) by default. The liquid inlet area was $8.3\;{\text{cm}}^{2}$, and the max liquid volume of the model was 97.2mL. The average airflow rate during breathing in a healthy individual is approximately 11L to 28L per minute [48]. Based on these considerations, the normal air flow velocity was set to 1m/s. And the swallowing process typically takes 1.2 to 1.7 seconds (from the entry of water into the mouth to its passage into the esophagus, with the residence time in the oral cavity being even shorter) [49]. However, a direct simulation of sequential mouthfuls of water would be impractical. Therefore, the drinking process was simulated as a continuous inflow. Considering the case of continuously drinking water, the normal velocity was set to 0.2m/s for water flow.

#### 2.1.4 Area of interest division

**Fig. 2:**
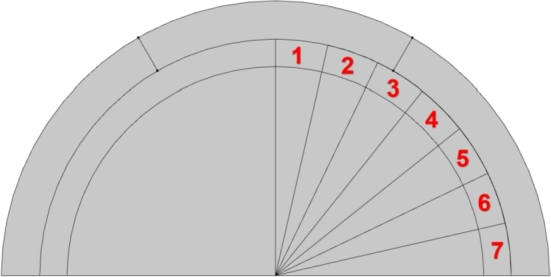
Area of interest division with each number reflecting a corresponding tooth.

As shown in Fig. 2, each half of the upper and lower jaws is evenly divided into seven sections to represent the approximate locations of different teeth in the mouth. The central incisor is labeled as 1, the lateral incisor as 2, the canine as 3, the first premolar as 4, the second premolar as 5, the first molar as 6, and the second molar as 7 [50]. The areas of interest include the facial (buccal) and lingual regions of teeth 1 to 7. [50], [51]. The facial region refers to the outer surface of the teeth facing the cheeks, while the lingual region refers to the inner surface facing the tongue.

The average temperature of the interested area is calculated as follows:

(5) $$T_{\text{area}}=\frac{\iint T\;dA}{\iint dA}$$

Where $T_{\text{area}}$ is the average temperature of the area, *T* is the temperature at each point in the area, and *dA* represents the area element.

#### 2.1.5 Mesh independence test

To ensure the accuracy of the simulation results and minimize computational cost, a mesh independence test was conducted using three different mesh densities: coarse mesh (number of elements: 10,598), medium mesh (number of elements: 27,639), and fine mesh (number of elements: 39,580). The analysis was performed by testing the average temperature of the facial and lingual surfaces of teeth regions 1 to 7 at t = 10 s, 30 s, and 60 s during the inflow of cold water at 6 °C (273.15 K) into the mouth with time step = 1 s.

As shown in Table 2a, the temperature deviations between the coarse and medium mesh were lower than 0.34%, while the deviations between the medium and fine mesh were lower than 0.05%. The results indicated that as the mesh was further refined, the relative error in temperature variation was reduced to less than 0.1%. Therefore, the medium mesh was chosen as the final meshing scheme to ensure a balance between calculation accuracy and computational efficiency.

**Table 2:** Mesh Density and Time Step Sensitivity Analysis.

Time (s)	Coarse	Medium (Percentage Deviation vs Coarse)	Fine (Percentage Deviation vs Medium)
10	307.52	307.99(0.15%)	307.99(0%)
30	304.47	305.29(0.27%)	305.24(0.02%)
60	301.93	302.97(0.34%)	302.82(0.05%)



Time (s)	0.1 s	0.5 s	1 s	5 s	10 s
10	307.10	307.14 (0.01%)	307.11 (0.00%)	307.14 (0.01%)	307.20 (0.03%)
30	304.41	304.45 (0.01%)	304.38 (0.01%)	304.43 (0.00%)	304.32 (0.03%)
60	302.29	302.30 (0.00%)	302.24 (0.02%)	302.31 (0.01%)	302.21 (0.03%)

Additionally, a time step sensitivity analysis was performed using the medium mesh, as shown in Table 2b, to evaluate the effect of different time step intervals on the results. The deviations for time steps of 0.1 s, 0.5 s, 1 s, 5 s, and 10 s were tested for time points 10 s, 30 s, and 60 s. The analysis showed that deviations remained within 0.03%, confirming that time step with 10 s has little effect on the results.

Considering that clinically accepted temperature sensors typically operate within an accuracy tolerance of ±0.2 °C, the minor differences observed between the medium and fine meshes fall well within this practical threshold. Therefore, the medium mesh was selected as the final meshing scheme, as it offers sufficient accuracy for capturing key thermal behavior patterns without incurring unnecessary computational cost.

### 2.2 Experimental study

This study was conducted with the approval of the Research Ethics Committee of the University of Oxford (R70833/RE001). Two healthy volunteers (a 27-year-old male and a 26-year-old female) were recruited for the experimental study. Before the tests, participants were asked to brush their teeth first, and their armpit temperatures were measured using a digital thermometer (DMT4132, Joytech Healthcare, Hangzhou, China), recording 37.1 °C and 36.8 °C, respectively.

#### 2.2.1 The effect of cold drinks on oral temperature

The experiment was conducted in an air-conditioned room with an ambient temperature of $22\pm 0.2\;{}^{\circ }$C, as measured by the thermometer (STH200, GEMlead, Fuzhou, China). Warm water was heated to 37 °C using a precision cooker (ISV-100W, Inkbird, Shenzhen, China). Before each trial, participants consumed 500 mL of warm water over a period of 5 minutes. Participants were free to either swallow or expel each sip after ensuring uniform distribution across the oral cavity.

The cold water used in the experiment was maintained at a constant temperature of 6 °C in a refrigerator. Each measurement session involved the consumption of 1 liter of cold water over a duration of 1 minute. Participants were free to either swallow or expel each sip after ensuring uniform distribution across the oral cavity.

To capture rapid temperature changes and minimize the thermal inertia associated with contact-based sensors, facial temperatures of the right upper and lower jaw teeth at positions 1, 3, and 5 were recorded using a non-contact infrared thermometer (K7100, OMRON, Shenzhen, China). Due to the line-of-sight requirement of infrared thermometry and anatomical constraints, it was not feasible to measure temperatures on lingual surfaces or on the facial side of posterior teeth (tooth 7). Therefore, the experimental measurements focused on accessible anterior and premolar facial surfaces, where stable and repeatable temperature readings could be reliably obtained.

Temperature measurements were taken at 20, 40, and 60 seconds during the one-minute period. The timer was paused during each measurement and resumed immediately afterward until the full minute was completed. In each session, only the temperature of a single specific location (e.g., the facial temperature of maxillary tooth 1) was recorded, with three repeated measurements taken at each site. Each participant completed six trials. Mouth breathing was strictly prohibited throughout the experiment.

#### 2.2.2 The effect of breathing frequency on oral temperature during mouth breathing

A breathing rate guidance video was made in advance to guide participants to breathe through their mouths with a fixed period (P=1.5 s, 2 s, 3 s, 4 s) during the test. Participants will have time to familiarize themselves with the breathing rate before the test officially begins. Before each trial. Participants will rest in the indoor environment with an ambient temperature of $22\pm 0.2\;{}^{\circ }$C, and consumed 500 mL of 37 °C warm water over a period of 5 minutes.

The experiment was conducted in an outdoor condition with an ambient temperature of $1.4\pm 1.9\;{}^{\circ }$C. Facial temperatures of the right upper and lower jaw teeth at positions 1, 3, and 5, were recorded using the non-contact thermometer. Temperature measurements were taken at 60, 120, and 300 seconds during 300 s. The timer was paused during each measurement and resumed immediately afterward until the full 300 s was completed. Mouth breathing was strictly prohibited during the measurement. In each session, only the temperature of a single specific location was recorded, with three repeated measurements taken at each site for each breath period. Each participant completed 24 trials.

## 3 Results

### 3.1 Oral temperature changes during taking cold liquid: comparison of simulation, experiment and literature case study

**Fig. 3:**
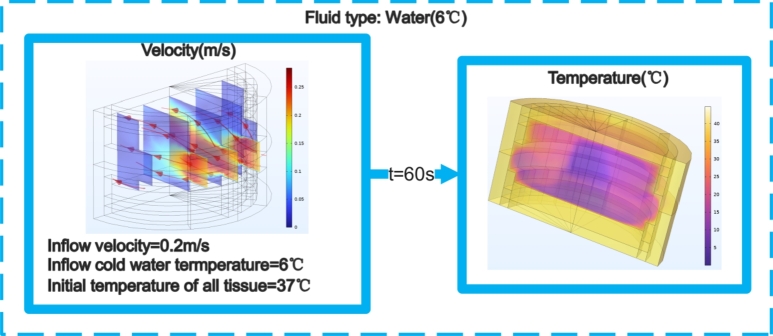
Simulation Procedure Overview: The total simulation time is 60 seconds, with an inflow cold water velocity of 0.2 m/s and a temperature of 6 °C. The initial temperature of all tissues is set to 37 °C.

Fig. 3 illustrates the simulation setup. In order to simplify the flow process, we set the flow condition of the model to laminar flow, the continuous cold water inflow velocity to 0.2 m/s, and the cold water continued to flow into the mouth for 60 s.

Apart from the computational and experimental studies, the data from a published case study was utilized for comparison. Temperature changes across various regions of the mouth after hot and cold stimuli, using thermocouples, was measured for one healthy volunteer [51].

The procedure involved consuming 250 ml of hot liquid at an initial temperature of 72.5 °C, sipped over 7 minutes (12 to 13 sips). After a 5-minute equilibration period, a cold liquid at an initial temperature of 6.0 °C was consumed over 1 minute in 6 sips. This process was repeated three times for each tooth pair measurement. Minimum temperatures (± SD) were recorded 12 times on the facial and lingual surfaces of the upper and lower central incisors, canines, second premolars, and second molars—corresponding to areas 1, 3, 5, and 7 in the model, respectively.

**Fig. 4:**
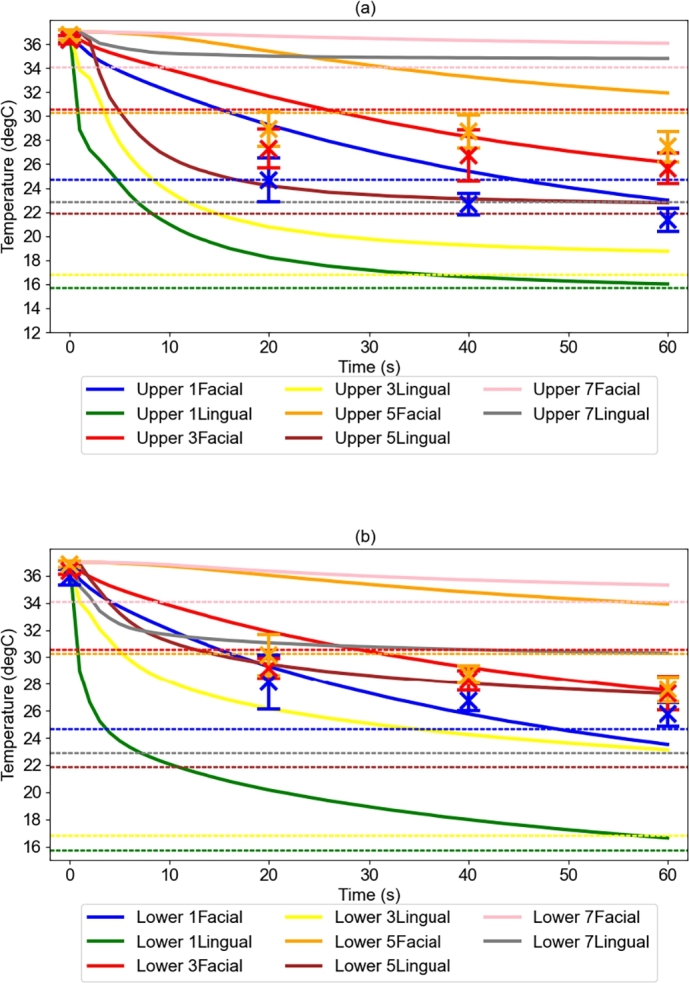
Comparison of the computational, experimental, and case study Results for the Upper Jaw (a) and Lower Jaw (b): Solid lines represent the simulated results, while dotted lines indicate the case study results. The experimental results are denoted by x markers: The anterior region shows greater temperature variation relative to the posterior region.

Computational, experimental, and case study results are illustrated in Fig. 4. Experimental values are presented as mean ± standard deviation (SD). Table 3 summarizes the quantitative comparisons between the simulated temperatures, experimental measurements, and case study data.

**Table 5:** Comparison of simulation, experimental, and case study temperatures results.

Upper Location	1 Facial	1 Lingual	3 Facial	3 Lingual	5 Facial	5 Lingual	7 Facial	7 Lingual	Overall

**Simulation**	22.99	16.02	26.14	18.74	31.92	22.78	36.06	34.80	–
**Experiment (SD)**	21.4 (1.0)	–	25.7 (1.3)	–	27.5 (1.3)	–	–	–	–
**Case Study (SD)**	24.70 (0.50)	15.70 (0.20)	30.60 (0.40)	16.80 (0.20)	30.30 (0.40)	21.90 (1.20)	34.10 (0.80)	22.90 (1.50)	–
**MD (Simulation vs. Case study)**	-1.71	0.32	-4.46	1.94	1.62	0.88	1.96	11.90	**1.56**

Lower Location	1 Facial	1 Lingual	3 Facial	3 Lingual	5 Facial	5 Lingual	7 Facial	7 Lingual	Overall

**Simulation**	23.52	16.63	27.51	23.12	33.92	27.29	35.32	30.30	–
**Experiment (SD)**	25.8 (0.9)	–	27.3 (1.3)	–	27.6 (1.0)	–	–	–	–
**Case Study (SD)**	30.90 (0.30)	30.00 (2.10)	25.80 (0.70)	31.50 (0.60)	30.00 (1.40)	34.20 (0.70)	34.40 (1.20)	34.20 (0.20)	–
**MD (Simulation vs. Case study)**	-7.38	-13.37	1.71	-8.38	3.92	-6.91	0.92	-3.90	**-4.17**



Upper Location	1 Facial	3 Facial	5 Facial

0 s	0.67/0.62/1.70%	0.72/0.65/1.80%	0.43/0.38/1.04%
20 s	4.89/4.59/19.18%	4.59/4.35/16.28%	6.58/6.45/22.55%
40 s	2.85/2.73/12.21%	2.51/1.88/7.54%	4.73/4.56/16.12%
60 s	1.87/1.64/7.88%	1.28/1.08/4.22%	4.60/4.45/16.43%
**Overall RMSE/MAE**	**4.52/3.39/13.46%**		

Lower Location	1 Facial	3 Facial	5 Facial

0 s	1.20/1.08/3.04%	0.67/0.63/1.75	0.29/0.22/0.59%
20 s	2.15/1.62/6.09%	2.80/2.72/9.39%	6.03/5.87/19.72%
40 s	1.22/1.09/4.01%	1.20/1.00/3.60%	6.11/6.08/21.24%
60 s	2.37/2.23/8.57%	1.16/0.99/3.65%	6.39/6.34/23.08%
**Overall RMSE/MAE**	**3.95/2.91/10.35%**		

Table 3a presents the temperature values at t = 60 s across facial and lingual positions in both the upper and lower jaws, along with the mean differences (MD) between the simulation and case study data.

Table 3b provides a detailed temporal validation by reporting the Root Mean Square Error (RMSE), Mean Absolute Error (MAE) and Mean Absolute Percentage Error (MAPE) between the simulated and experimental temperatures at 0 s, 20 s, 40 s, and 60 s for selected facial positions (1, 3, and 5) in both jaws. The overall RMSE and MAE values reflect the cumulative agreement across all time points and positions

**Fig. 5:**
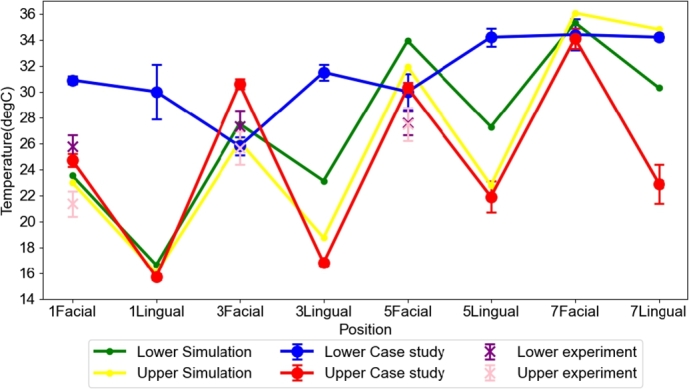
Simulation temperature at t=60 s compared with experimental temperature: Significant facial-lingual temperature differences are observed, except at lower jaw locations in the case study.

For the upper locations, simulated temperatures at t = 60 s ranged from 16.02 °C at 1 Lingual to 36.06 °C at 7 Facial. Experimental values were available for selected facial positions, showing temperatures between 21.4 °C (1 Facial) and 27.5 °C (5 Facial). Case study values exhibited a broader range, with the lowest recorded temperature of 15.70 °C at 1 Lingual and the highest at 34.10 °C at 7 Facial. See Fig. 5.

For the lower locations, simulated temperatures varied from 16.63 °C at 1 Lingual to 35.32 °C at 7 Facial. Experimental temperatures showed a narrower range, from 25.8 °C (1 Facial) to 27.6 °C (5 Facial). In contrast, case study values displayed higher variability, with temperatures spanning from 25.80 °C at 3 Facial to 34.40 °C at 7 Facial. Compared to the upper locations, the case study data for the lower jaw did not exhibit a clear temperature differentiation between the lingual and facial areas. In contrast, the simulation results consistently demonstrated distinct temperature patterns between lingual and facial surfaces in both the upper and lower jaws.

The simulation results were compared against the case study data using the reported mean values. For the upper locations, the RMSE, MAE, and MAPE were 4.68 °C, 3.10 °C, and 12.77%, respectively. For the lower locations, these metrics were higher, with an RMSE of 6.94 °C, MAE of 5.81 °C, and MAPE of 18.63%.

In addition, the simulation results were compared with the experiment results. Compared with the experimental results, the overall RMSE and MAE (MAPE) for the upper facial positions were 4.52 °C and 3.39 °C (13.46%), respectively. For the lower facial positions, these values were slightly lower at 3.95 °C and 2.91 °C (10.35%).

### 3.2 Oral temperature of simulated and experiment results

#### 3.2.1 Oral temperature during continuously breathing cold air (0 °C)

This simulation models temperature changes in the oral cavity during continuous breathing of cold air over an extended period. Each breathing cycle has a duration of P seconds, consisting of P/2 seconds of inhaling 0 °C cold air, followed by P/2 seconds of exhaling 37 °C air, where P is the period of one complete breathing cycle. The air inflow speed is set to 1 m/s.

**Fig. 6:**
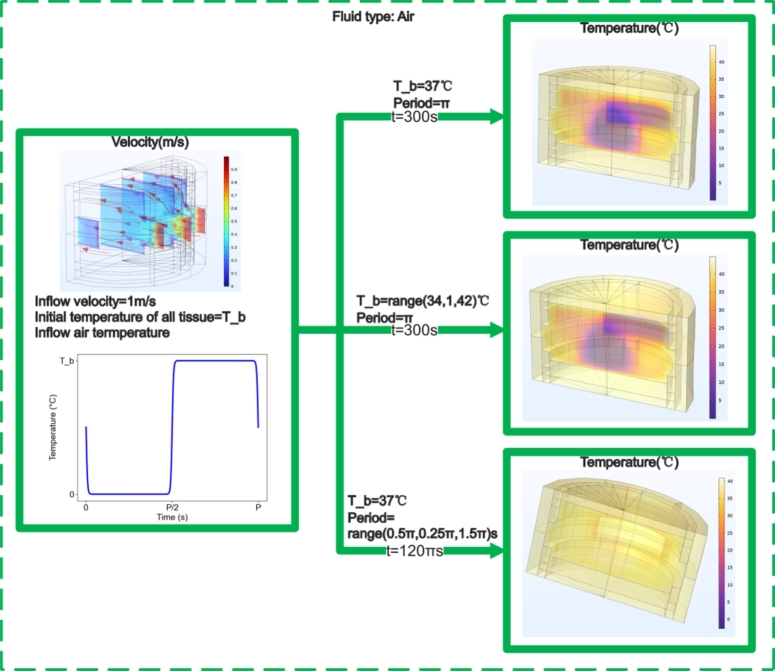
Simulation Procedure Overview: The inflow air temperature follows a rectangular wave function with a period *P* and an inflow velocity of 1 m/s. The initial tissue temperature is set to *T*_*b*_, representing the blood (core) temperature. Three simulation scenarios were conducted: (1) *T*_*b*_ = 37 ^∘^C,Period = *π* s: This scenario simulates the response of oral temperature over 300 seconds of simulation time. (2) *T*_*b*_ ∈ range(34 ^∘^C,1 ^∘^C,42 ^∘^C),Period = *π* s: This scenario explores the effect of varying blood temperatures on the response of oral temperature after 300 seconds of simulation time. (3) *T*_*b*_ = 37 ^∘^C,Period ∈ range(0.5*π*,0.25*π*,1.5*π*) s: This scenario investigates the impact of different breathing rates (represented by varying periods) on the response of oral temperature after 120*π* seconds.

As shown in Fig. 6, the exhaled warm air's inflow direction was aligned with that of the inhaled air to avoid disturbances in the velocity field and to maintain stable boundary conditions at the inlet and outlet. This setup prevents instability that could result from reversing the airflow during exhalation.

**Fig. 7:**
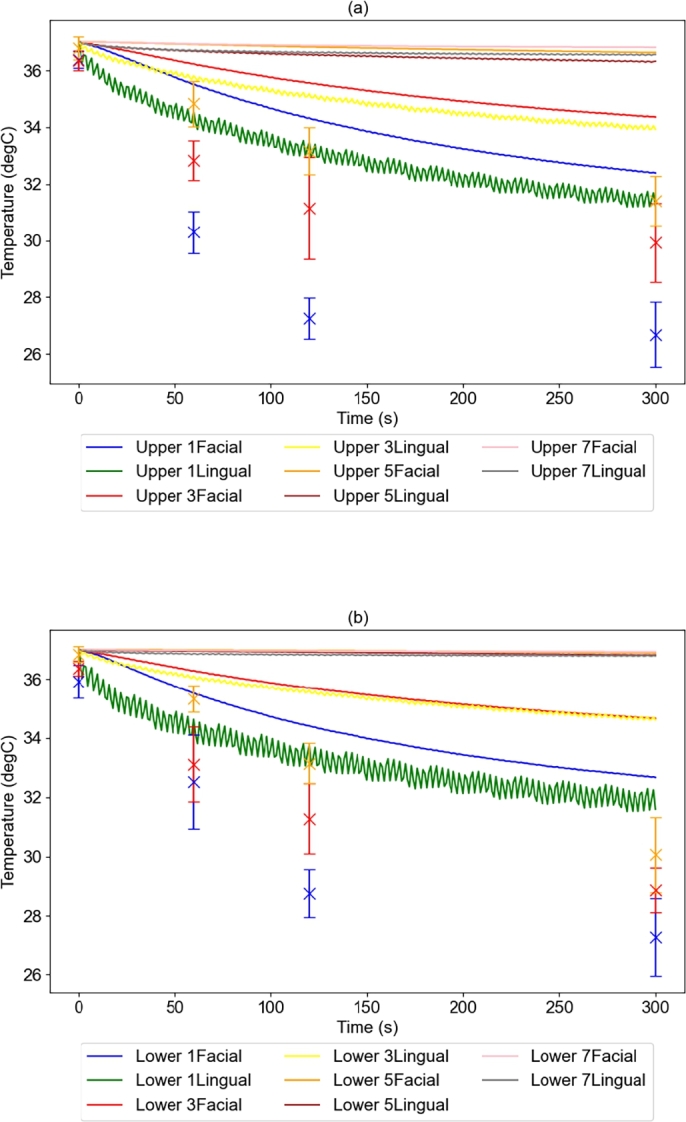
Simulation results (P = *π* s) and experimental results (P=3 s) during continuously breathing cold air (Upper jaw(a) and Lower jaw (b)). The simulation results and experimental results are denoted by lines and x markers, respectively: The anterior region shows greater temperature variation relative to the posterior region.

Fig. 7 illustrates the temperature changes over time during simulations, with a respiratory cycle of P = *π* seconds, and experimental temperature variations over time with a respiratory cycle of P = 3 seconds. The total duration was 300 seconds, and the blood (core) temperature was set at 37 °C. Overall, temperatures gradually decrease over time, with the extent of change varying across different locations.

In the simulation results, temperature drops are most pronounced in the anterior tooth area, where both 1 Facial and 1 Lingual show significant decreases. This suggests that anterior teeth are more affected by continuous exposure to cold air, leading to a rapid temperature drop. In contrast, temperatures at middle and posterior positions are more stable, with gentler decreases over time. For instance, temperatures at 5 Facial and 7 Facial are higher and less affected, indicating that posterior teeth are less influenced by cold air exposure. Additionally, buccal temperatures are typically higher and more stable than lingual temperatures at the same location. For example, the temperature at 3 Facial remains consistently higher than at 3 Lingual.

The experimental results confirm that in the facial area, temperature changes follow the same trend; however, the temperatures are lower than the simulation results.

**Fig. 8:**
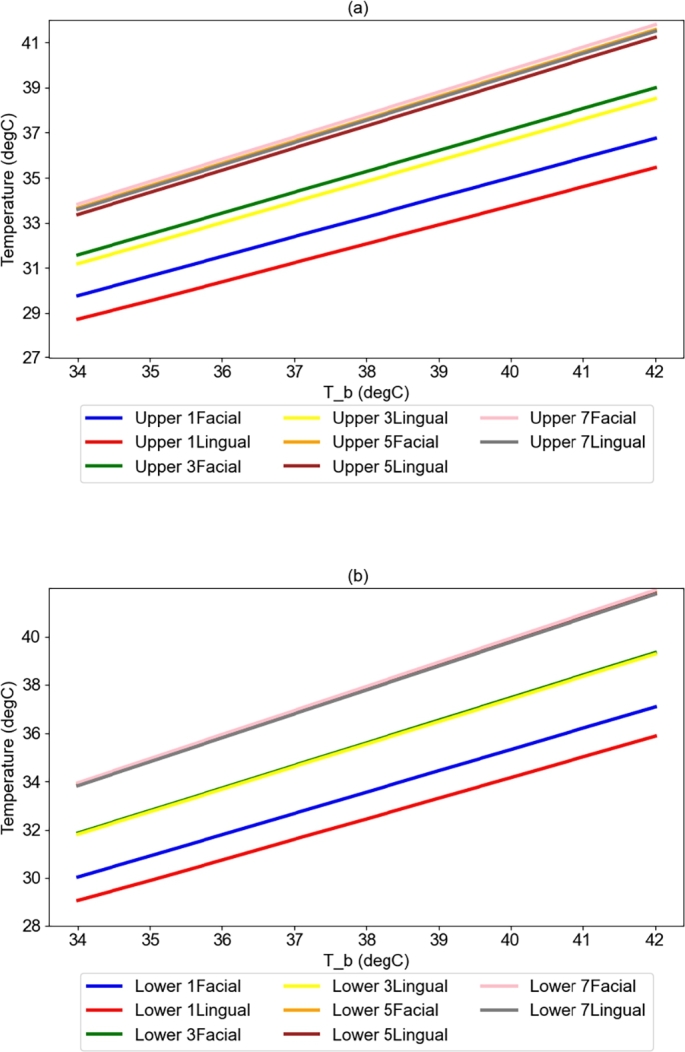
Relationship between blood temperature (*T*_*b*_) and location temperature after 5 min of repeated exposure to cold air on the oral cavity (Upper jaw(a) and Lower jaw (b)): The temperature at each location is positively correlated with the core temperature.

Fig. 8 examines the relationship between oral temperatures and blood (core) temperature at P=*π* seconds and t=300 seconds of the simulation results, to assess the long-term thermal effects of repeated cold air exposure. The core temperature varies from 34 °C to 42 °C, with temperatures at each oral location increasing as core temperature rises, showing a positive correlation. At a low core temperature (e.g., 34 °C), anterior regions exhibit lower temperatures, while posterior regions remain warmer. As core temperature increases, temperatures in all regions of the maxilla and mandible also rise, with anterior-posterior temperature differences persisting. Generally, posterior regions maintain higher temperatures than anterior regions, suggesting that posterior locations respond more significantly to changes in core temperature.

**Fig. 9:**
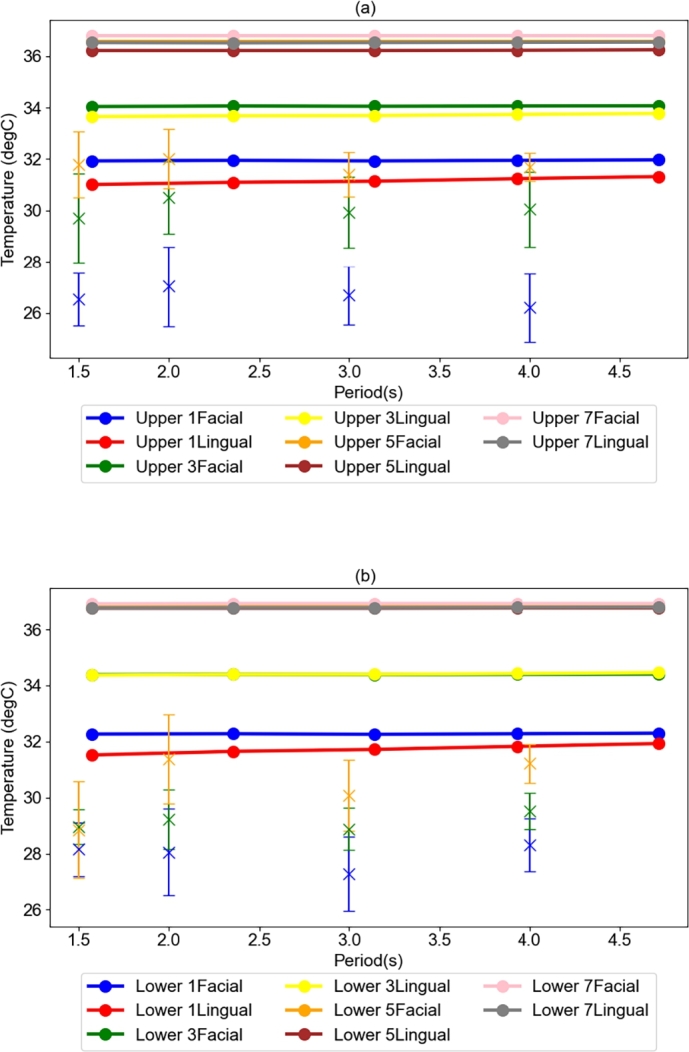
Relationship between respiration rate and location temperature after 5 min of repeated exposure to cold air on the oral cavity (Upper jaw(a) and Lower jaw (b)). Simulation results are represented by o markers with lines, while experimental results are indicated by x markers: Respiration cycle length has minimal impact on oral temperature.

Fig. 9 shows the relationship between oral temperatures and respiration period of both computational and experimental results, with core temperature set to 37 °C and t=120*π* seconds for the simulation, to assess the long-term thermal effects of respiration rate on the oral cavity. Overall, respiration cycle length has minimal impact on oral temperature.

#### 3.2.2 Oral temperature recovery after taking cold liquid

**Fig. 10:**
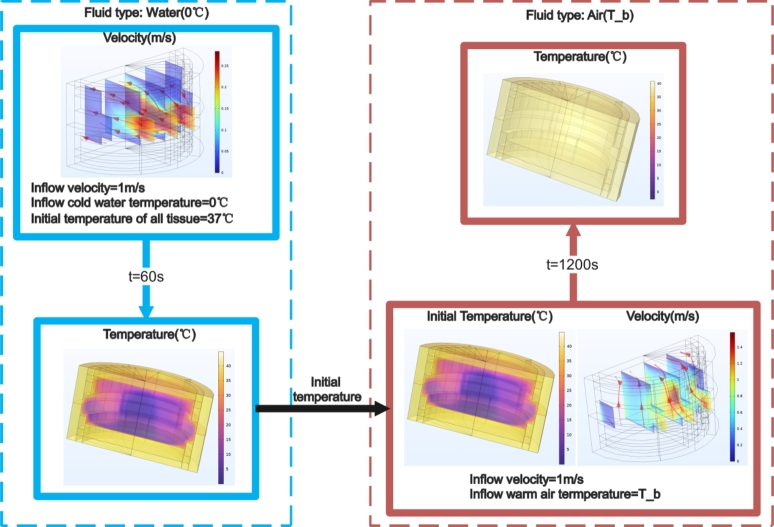
Simulation Procedure Overview: 0 °C cold water was simulated to flow into the oral cavity at a speed of 0.2 m/s over 60 seconds, then there was the inhalation of 37 °C air at an inflow speed of 1 m/s for 600 seconds.

One challenge in oral temperature measurement is the recovery time after ingesting cold liquids. Using the proposed computational model, the temperature recovery process following cold liquid intake was simulated. Fig. 10 illustrates this simulation. To establish initial thermal conditions, 0 °C cold water was simulated to flow into the oral cavity at a speed of 0.2 m/s over 60 seconds. Afterward, the simulation continued with inhalation of 37 °C air at an inflow speed of 1 m/s for 600 seconds. Inhaling warm air rather than exhaling was chosen to simplify the velocity field and boundary conditions, thus avoiding disruptions that exhaled air could introduce to the fluid flow pattern at the inlet and outlet.

**Fig. 11:**
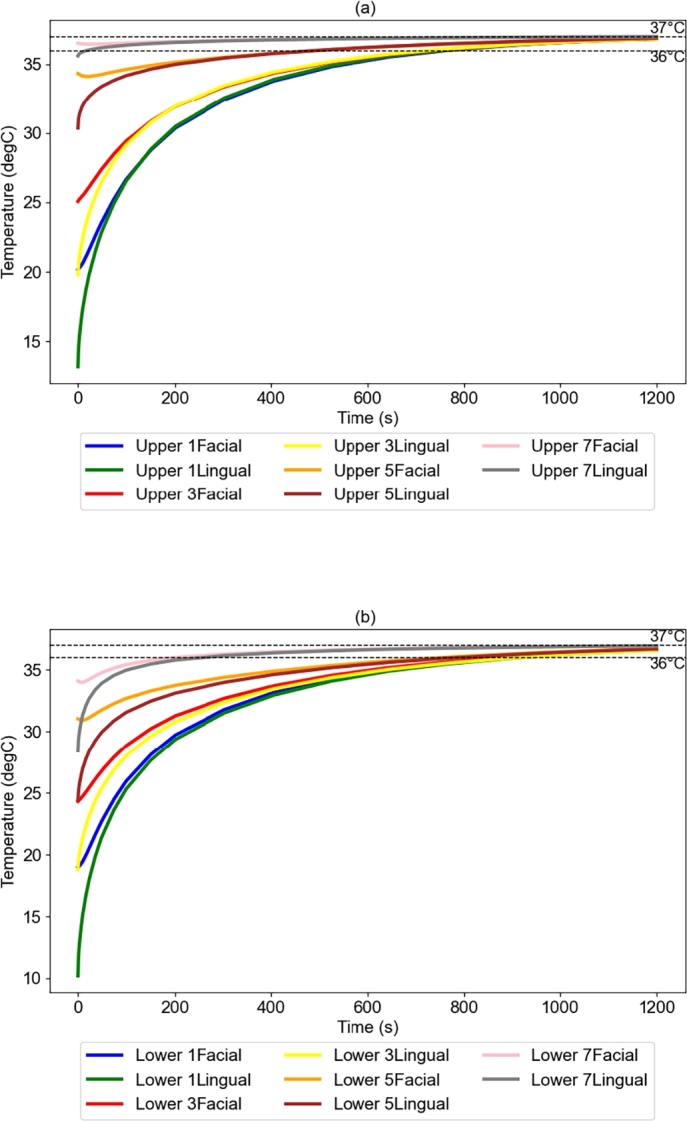
Simulated temperatures recovery process after taking 0 °C cold liquid (Upper jaw(a) and Lower jaw (b)): Most of locations returned to within 1 °C of core temperature within 15 minutes.

Fig. 11 presents the temperatures of the facial and lingual surfaces of teeth 1, 3, and 7 in both the upper and lower jaws during inhalation of warm air. After cold water intake, temperatures at all locations gradually increased over time with continuous inhalation of warm air, approaching the core temperature of 37 °C. The anterior tooth region, particularly affected by the cold water, showed a longer recovery period, while the posterior teeth were less impacted and recovered more quickly. Most of locations returned to within 1 °C of core temperature within 900 seconds (15 minutes).

## 4 Discussion

Both simulation and experimental results highlight distinct thermal responses across different regions of the oral cavity, with significant variations between anterior and posterior teeth. The anterior teeth exhibited greater sensitivity to external temperature changes, such as cold water intake and cold air exposure, whereas the posterior teeth maintained greater thermal stability. Additionally, the simulation results indicate that anterior teeth recover more slowly after exposure to cold stimuli, while posterior teeth retain higher temperatures and return to equilibrium more quickly. These findings suggest that the posterior regions of the oral cavity are less affected by environmental cooling.

These differences can be attributed to fundamental heat transfer principles and anatomical features. While soft tissue thickness is same across regions during simulation, the anterior teeth are surrounded by a smaller soft tissue area compared to posterior teeth. This limited surface area reduces the potential for heat replenishment via perfusion from surrounding tissues and blood flow. In addition, the actual anatomical structure, the anterior teeth not only have a smaller soft tissue area, but also a lower actual blood perfusion rate [52], [53]. Furthermore, the cold fluid is gradually warmed as it flows toward the posterior, the anterior teeth experience the most pronounced initial cooling. In contrast, the posterior region benefits from a better soft tissue coverage and richer vascularization, providing better thermal insulation and more efficient heat recovery following cold exposure.

Furthermore, the simulation and experiment, and the cited case study data consistently showed that buccal surfaces exhibit higher and more stable temperatures compared to lingual surfaces on the upper location. This buccal-lingual temperature difference was typically larger than 5 °C. Considering that most medical-grade temperature sensors offer accuracy within ±0.2 °C, the differences between buccal and lingual regions are substantial and must be factored into sensor placement and calibration to ensure reliable core temperature estimation.

On the upper jaw, the buccal side generally benefits from greater insulation due to its proximity to the cheeks and less direct exposure to airflow or ingested fluids. In contrast, the lingual surfaces are more exposed to the fluid. This leads to consistently lower temperatures on the lingual side.

Another key finding of this study is that during mouth breathing in a cold environment, the total duration of exposure significantly influences oral temperature, whereas breathing frequency has only a minimal effect within normal physiological ranges. Both simulation and experiment results demonstrate that, under identical exposure durations, the exposure time strongly impacts oral temperatures across all regions, while changes in respiratory rate produce negligible differences. In addition, the simulation results show that both anterior and posterior oral regions exhibit proportional temperature rises as core temperature increases. These observations suggest that, despite external cooling, the oral cavity maintains a relatively stable thermal response, making it a potential site for monitoring core temperature changes in extreme conditions.

These findings have important implications for the development of the data driven sensor calibration and temperature estimation algorithms in wearable biosensors. Recognizing that long-term temperature variations are predominantly driven by exposure duration rather than breathing frequency, especially in thermally stable regions (posterior buccal area), data driven calibration algorithms can be designed to better filter out environmental disturbances. Compensation strategies can focus on managing the effects of prolonged cold exposure, avoiding unnecessary adjustments for normal variations in respiratory rate.

It is important to note that the simulated temperatures were higher than the experimental results under both cold water intake and cold air exposure conditions. Overall, the simulation results deviated from the experimental results by 10% to 13%. In addition to geometric differences between the model and the actual oral cavity, several factors may contribute to these discrepancies: 1. The simulation does not account for tongue movement, which can influence liquid flow within the oral cavity. 2. In the simulation, the exhaled air temperature is set to the core body temperature, whereas in reality, the temperature of the exhaled air is typically lower than this value. 3. The presence of saliva, which plays a crucial role in heat dissipation through evaporation, is not considered in the model. The absence of these factors may result in an overestimation of oral temperatures in the simulation.

Compared with the cited case study, our computational model shows considerable differences in the temperature distribution in some areas, especially the lower jaw region. For example, in the experimental results, the temperature difference between the buccal and lingual sides of the upper jaw is obvious, while in the lower area, the temperature difference between the buccal and lingual sides is not obvious. This difference may be related to the role of the tongue during swallowing. Normally, the swallowing action is mainly initiated by the tongue, which plays a certain protective role in the mandibular area [28], [54]. Furthermore, swallowing patterns vary among individuals [55], [56]. Different individuals often exhibit different temperature trends reported in the literature [50], [51]. In addition, variations in measurement equipment can contribute to differences in recorded temperatures, especially with contact sensors. When measuring oral temperature using thermocouples or thermistors, the sensor is typically placed on the surface rather than inside the teeth or underneath the mucosa. As a result, when cold liquid enters the mouth, it first comes into contact with the sensor before reaching the underlying oral tissue. Meanwhile, these contact sensors require a certain response time to register temperature changes accurately. All these factors can influence the experimental outcomes, which need to be taken into account when we consider the temperatures obtained from our model and experiment.

Our finite element model serves as a tool for simulating heat conduction and temperature distribution within the oral cavity. While some discrepancies exist between the model and specific experimental data, it effectively captures temperature trends across various regions of the oral cavity. This capability enables temperature trends predictions across a wide range of environmental conditions without being constrained by individual variability.

In the future, this model can serve as a powerful supplement to experimental research and provide theoretical support for the optimal design of temperature monitoring equipment. By predicting the oral temperature distribution under different conditions, this model can provide support for the placement of sensors in complex environments, especially when experiments are difficult or costly. Beyond device development, the model can provide valuable insights for clinical applications. For instance, it could support research on oral health conditions where temperature variations play a role, such as in assessing thermal sensitivity of dental tissues or studying drug dissolution rates in temperature-sensitive drug delivery methods [57], [58].

Furthermore, while this study identifies posterior oral regions as favorable sites for temperature monitoring due to their thermal stability and faster recovery to core temperature, it is important to consider the role of sensor response speed in practical applications. Sensors with significant thermal inertia may introduce delays or dampen transient temperature changes, particularly in regions where rapid cooling or recovery occurs, such as the anterior teeth. In contrast, the relatively stable thermal environment of posterior regions may mitigate the impact of sensor latency, enhancing measurement reliability. Future studies could incorporate sensor response characteristics into computational models to optimize both anatomical placement and device performance for real-time oral temperature monitoring.

The model also has certain limitations. Notably, it assumes static oral structures throughout both breathing and swallowing processes. In reality, the tongue, cheeks, and soft palate dynamically adjust their positions, continuously altering the geometry of the oral cavity and influencing airflow pathways, fluid distribution, and localized heat transfer dynamics.

Previous studies using tagged magnetic resonance imaging (MRI) have demonstrated that the genioglossus muscle exhibits rhythmic anterior-posterior movements during quiet breathing, with displacements of approximately 1 mm and local strains reaching up to 17%, indicating substantial tissue deformation even under normal respiratory conditions [59]. In addition, during swallowing, the tongue actively guides fluid flow through coordinated movements of both intrinsic and extrinsic muscles, which work together to compress the oropharynx and propel the bolus while shielding specific regions (e.g., mandibular lingual surfaces) from direct exposure to cold stimuli [28], [54], [60]. These complex muscular actions not only ensure effective bolus transport but also modulate fluid distribution within the oral cavity, thereby influencing localized heat transfer dynamics. These dynamic adjustments, along with subtle movements of the cheeks and soft palate during breathing or swallowing, can modulate airflow velocity profiles, contact areas, and thermal boundary layers, thereby directly impacting transient temperature distributions within the oral cavity.

By neglecting these dynamic movements, the current model may underestimate localized cooling effects, over-simplify thermal gradients, and reduce the accuracy of predicted heat transfer, particularly in regions where tissue motion provides protective or redistributive functions. This limitation likely contributes to the observed discrepancies between simulated and experimental temperatures in specific areas.

Another limitation of the model is the assumption of a constant arterial blood temperature across all tissue boundaries. This simplification was made to reduce computational complexity and is commonly adopted in bioheat thermal simulations [34], [61]. Given that this study focuses on short-term thermal responses to cold stimuli, systemic arterial temperature fluctuations are expected to be minimal under normal physiological conditions.

However, this assumption neglects the fact that arterial blood does not maintain core temperature throughout its circulation [62], [63]. As blood travels from the core to peripheral regions, including the oral cavity, it can undergo gradual cooling due to heat exchange with surrounding tissues, especially under cold exposure conditions. In regions with richer perfusion, such as the posterior oral cavity, this localized decrease in arterial blood temperature may limit the capacity for heat replenishment. Consequently, the model could overestimate tissue temperatures in these highly perfused areas, leading to an underestimation of the actual cooling effect, particularly in the posterior regions where blood flow plays a significant role in thermal regulation. Future work could incorporate dynamic blood temperature modeling or perfusion-adaptive boundary conditions to enhance accuracy, especially in scenarios involving prolonged or extreme cold exposure.

Additionally, the current model lacked evaporative cooling effects from saliva and mucosal surfaces. Evaporation is a well-established mechanism of heat loss in the oral cavity, particularly during mouth breathing when airflow promotes moisture evaporation from wet tissues. By neglecting this factor, the model likely overestimates oral temperatures during respiratory simulations, especially in regions with extensive mucosal exposure such as lingual areas. This simplification was made to avoid the added complexity of modeling coupled heat and mass transfer, which would require detailed characterization of saliva distribution, local humidity dynamics, and airflow-surface interactions. While this approach is reasonable for cold water intake scenarios—where the presence of liquid suppresses evaporation—the absence of evaporative cooling in breathing simulations may reduce accuracy, particularly under prolonged exposure or in low-humidity environments. Future work should consider incorporating simplified evaporation models to better capture these thermodynamic effects.

Finally, the experimental validation in this study was conducted with a limited sample size of two healthy volunteers, both within a narrow age range. This inherently restricts the generalizability of the experimental findings due to potential variability in oral anatomy, saliva production, breathing patterns, and individual thermal responses across broader populations. The computational model also assumes average anatomical and physiological conditions, without accounting for inter-individual differences such as variations in oral cavity geometry, tissue properties, or perfusion rates. Future work should incorporate sensitivity analyses and subject-specific modeling approaches to enhance the generalizability and personalized applicability of the findings.

## 5 Conclusion

The findings of this study provide valuable insights into the transient heat transfer processes in the oral cavity during cold fluid intake and cold air inhalation. A finite element model was developed to simulate thermal behavior under various conditions, including cold water ingestion, alternating exposure to hot and cold air, and temperature recovery after cold water intake through hot air inhalation. Additionally, experimental measurements were conducted to capture the actual thermal response of the oral cavity to these transient heat transfer processes.

Both simulation and experiment results showed that the total duration of mouth breathing significantly affects oral temperature, while variations in the respiratory cycle have minimal effect. The simulation results also indicated that after cold stimulation, anterior teeth experience slower temperature recovery, while posterior teeth regions remain more stable and at relatively higher temperatures. Most areas of the oral cavity returned to near-core temperature within 15 minutes.

With the growing adoption of wearable biosensors for non-invasive core temperature estimation, this study offers critical guidance for optimizing sensor placement and informing the development of data-driven calibration algorithms to mitigate environmental thermal disturbances. These insights are particularly relevant for applications in sports medicine, continuous fever monitoring, occupational health, and remote patient care, where accurate core temperature tracking is essential under dynamic conditions.

Future research should focus on enhancing the model's physiological realism by incorporating factors such as dynamic saliva distribution, evaporative cooling, humidity variations, and tissue movement during breathing and swallowing. Moreover, extending the simulations to include diverse scenarios such as varying airflow rates and intermittent fluid intake patterns would improve applicability. Investigating inter-individual anatomical differences through subject-specific modeling could further refine temperature sensor design and calibration strategies.

## Declaration of generative AI and AI-assisted technologies in the writing process

During the preparation of this work the author(s) used ChatGPT 4o in order to refine sentence structure and enhance readability. After using this tool/service, the author(s) reviewed and edited the content as needed and take(s) full responsibility for the content of the published article.

## Funding

This work was supported by the 10.13039/501100000268Biotechnology and Biological Sciences Research Council (BBSRC), grant number UKRI012, and by the National Institute for Health and care Research (NIHR) HealthTech Research Centre for Community Healthcare at Oxford Health NHS Foundation Trust. The views expressed are those of the author(s) and not necessarily those of the NHS, the NIHR or the Department of Health and Social Care.

## CRediT authorship contribution statement

**Yuanzhe Zhao:** Writing – original draft, Visualization, Validation, Software, Methodology, Formal analysis, Data curation, Conceptualization. **Jeroen H.M. Bergmann:** Writing – review & editing, Validation, Supervision, Project administration, Methodology, Funding acquisition, Formal analysis, Conceptualization.

## Declaration of Competing Interest

The authors declare no conflict of interest.

## Data Availability

Data will be made available on request.

## References

[1] Campbell I. (2008). Body temperature and its regulation. Anaesth. Intensive Care Med..

[2] Ogoina D. (2011). Fever, fever patterns and diseases called ‘fever’–a review. J. Infect. Public Health.

[3] Yamamoto S. (2016). Body temperature at the emergency department as a predictor of mortality in patients with bacterial infection. Medicine.

[4] Laupland K.B. (2012). Determinants of temperature abnormalities and influence on outcome of critical illness. Crit. Care Med..

[5] Racinais S. (2015). Consensus recommendations on training and competing in the heat. Scand. J. Med. Sci. Sports.

[6] Henderson M.J., Grandou C., Chrismas B.C.R., Coutts A.J., Impellizzeri F.M., Taylor L. (2023). Core body temperatures in intermittent sports: a systematic review. Sports Med..

[7] Leon L., Kenefick R. (2012). Textbook of Wilderness Medicine.

[8] Lim C.L., Byrne C., Lee J.K. (2008). Human thermoregulation and measurement of body temperature in exercise and clinical settings. Ann. Acad. Med. Singap..

[9] Fortney S.M., Vroman N.B. (1985). Exercise, performance and temperature control: temperature regulation during exercise and implications for sports performance and training. Sports Med..

[10] Moran D.S., Mendal L. (2002). Core temperature measurement. Sports Med..

[11] Hymczak H. (2021). Core temperature measurement—principles of correct measurement, problems, and complications. Int. J. Environ. Res. Public Health.

[12] Niedermann R., Wyss E., Annaheim S., Psikuta A., Davey S., Rossi R.M. (2014). Prediction of human core body temperature using non-invasive measurement methods. Int. J. Biometeorol..

[13] Ganio M.S., Brown C.M., Casa D.J., Becker S.M., Yeargin S.W., McDermott B.P., Boots L.M., Boyd P.W., Armstrong L.E., Maresh C.M. (2009). Validity and reliability of devices that assess body temperature during indoor exercise in the heat. J. Athl. Train..

[14] Casa D.J., Becker S.M., Ganio M.S., Brown C.M., Yeargin S.W., Roti M.W., Siegler J., Blowers J.A., Glaviano N.R., Huggins R.A., Armstrong L.E., Maresh C.M. (Jul.–Sep. 2007). Validity of devices that assess body temperature during outdoor exercise in the heat. J. Athl. Train..

[15] Dolson C.M., Harlow E.R., Phelan D.M., Gabbett T.J., Gaal B., McMellen C., Geletka B.J., Calcei J.G., Voos J.E., Seshadri D.R. (2022). Wearable sensor technology to predict core body temperature: a systematic review. Sensors.

[16] Han X., Wu J., Hu Z., Li C., Hu X. (2025). A practical deep learning model for core temperature prediction of specialized workers in high-temperature environments. J. Therm. Biol..

[17] Hamatani T., Uchiyama A., Higashino T. (2015). Proceedings of the 2015 ACM International Joint Conference on Pervasive and Ubiquitous Computing.

[18] Li Y., Tang H., Liu Y., Qiao Y., Xia H., Zhou J. (2022). Oral wearable sensors: health management based on the oral cavity. Biosens. Bioelectron. X.

[19] de Almeida e Bueno L., Milnthorpe W., Bergmann J.H.M. (2022). Determining the performance of a temperature sensor embedded into a mouthguard. BDJ Open.

[20] de Almeida e Bueno L., Kwong M.T., Bergmann J.H.M. (2023). Performance of oral cavity sensors: a systematic review. Sensors.

[21] Cheng R., Bergmann J. (2023). A case for an oral cavity-based respiratory rate sensor system. IEEE Sens. Lett..

[22] Mazerolle S.M., Ganio M.S., Casa D.J., Vingren J., Klau J. (2011). Is oral temperature an accurate measurement of deep body temperature? A systematic review. J. Athl. Train..

[23] Taylor N.A., Tipton M.J., Kenny G.P. (2014). Considerations for the measurement of core, skin and mean body temperatures. J. Therm. Biol..

[24] Zhao Y., de Almeida e Bueno L., Holdsworth D.A., Bergmann J.H.M. (2024). Evaluating the agreement between oral, armpit, and ear temperature readings during physical activities in an outdoor setting. Int. J. Environ. Res. Public Health.

[25] Stojanović G.M. (2024). Temperature sensors manufactured from edible materials intended for oral cavity operation. Int. J. Precis. Eng. Manuf. Green Technol..

[26] Kim J.J., Stafford G.R., Beauchamp C., Kim S.A. (2020). Development of a dental implantable temperature sensor for real-time diagnosis of infectious disease. Sensors.

[27] Iitani K. (2023). Wireless unconstrained monitoring of intra-oral temperature using thermistor and telemeter sealed in mouthguard. Sens. Mater..

[28] Barclay C.W., Spence D., Laird W.R.E. (2005). Intra-oral temperatures during function. J. Oral Rehabil..

[29] Airoldi G., Riva G., Vanelli M., Filippi V., Garattini G. (1997). Oral environment temperature changes induced by cold/hot liquid intake. Am. J. Orthod. Dentofac. Orthop..

[30] Palmer D.S., Barco M.T., Billy E.J. (1992). Temperature extremes produced orally by hot and cold liquids. J. Prosthet. Dent..

[31] Michailesco P.M., Marciano J., Grieve A.R., Abadie M.J.M. (1995). An in vivo recording of variations in oral temperature during meals: a pilot study. J. Prosthet. Dent..

[32] Xie H., Deng S., Zhang Y., Zhang J. (2017). Proceedings of the 8th IEEE International Conference on Software Engineering and Service Science (ICSESS).

[33] Arathy K., Sudarsan N., Antony L. (2022). Early detection and parameter estimation of tongue tumour using contact thermometry in a closed mouth. Int. J. Thermophys..

[34] Xu X.Y., Ni S.J., Fu M., Zheng X., Luo N., Weng W.G. (2017). Numerical investigation of airflow, heat transfer and particle deposition for oral breathing in a realistic human upper airway model. J. Therm. Biol..

[35] Kulkarni N.A., Kleinstreuer C. (2020). High-temperature effects on the mucus layers in a realistic human upper airway model. Int. J. Heat Mass Transf..

[36] Lin M., Liu Q.D., Xu F., Bai B.F., Lu T.J. (Apr. 14, 2010). Proceedings of the Fourth International Conference on Experimental Mechanics, Proc. SPIE 7522, 75222N.

[37] Sabaeian M., Shahzadeh M. (2015). Simulation of temperature and thermally induced stress of human tooth under CO_2_ pulsed laser beams using finite element method. Lasers Med. Sci..

[38] Zhu C., Xie H., Chen X., Wang X., Meng J., Wei J., Zhao Z. (2024). Numerical simulation on exhaled aerosol transmission based on realistic oral-nasal structures and temperature distribution. J. Air Waste Manage. Assoc..

[39] Dias P.E.M., Miranda G.E., Beaini T.L., Melani R.F.H. (2016). Practical application of anatomy of the oral cavity in forensic facial reconstruction. PLoS ONE.

[40] Amelot A. (2015). Anatomical features of skull base and oral cavity: a pilot study to determine the accessibility of the sella by transoral robotic-assisted surgery. Neurosurg. Rev..

[41] Bergman R.T. (1999). Cephalometric soft tissue facial analysis. Am. J. Orthod. Dentofac. Orthop..

[42] Tandon M., Singh H., Singla N., Jain P., Pandey C.K. (2020). Tongue thickness in health vs cirrhosis of the liver: prospective observational study. World J. Gastrointest. Pharmacol. Ther..

[43] Lin M., Xu F., Lu T.J., Bai B.F. (2010). A review of heat transfer in human tooth—experimental characterization and mathematical modeling. Dent. Mater..

[44] Lovik R.D., Abraham J.P., Sparrow E.M. (2009/07/01). Potential tissue damage from transcutaneous recharge of neuromodulation implants. Int. J. Heat Mass Transf..

[45] González-Suárez A., Pérez J.J., Berjano E. (2018/04/20). Should fluid dynamics be included in computer models of RF cardiac ablation by irrigated-tip electrodes?. Biomed. Eng. Online.

[46] González-Suárez A., Berjano E. (2016). Comparative analysis of different methods of modeling the thermal effect of circulating blood flow during RF cardiac ablation. IEEE Trans. Biomed. Eng..

[47] Singh S., Saccomandi P., Melnik R. (2022). Three-phase-lag bio-heat transfer model of cardiac ablation. Fluids.

[48] Zuurbier M., Hoek G., Hazel P.V.D., Brunekreef B. (2009). Minute ventilation of cyclists, car and bus passengers: an experimental study. Environ. Health.

[49] Dodderi T., Puthiry M., Thomas S. (2020). How much is too much? Effect of volume on water swallowing test. J. Nat. Sci. Biol. Med..

[50] Ernst C.-P., Canbek K., Euler T., Willershausen B. (2004). In vivo validation of the historical in vitro thermocycling temperature range for dental materials testing. Clin. Oral Invest..

[51] Youngson C., Barclay C. (2000). A pilot study of intraoral temperature changes. Clin. Oral Invest..

[52] Naumova E.A., Dierkes T., Sprang J. (2013). The oral mucosal surface and blood vessels. Head Face Med..

[53] Alessandro S.G., Antonino A., Messina P. (2009). Anatomical evaluation of oral microcirculation: capillary characteristics associated with sex or age group. Ann. Anat..

[54] Chen J. (2009/01/01). Food oral processing—a review. Food Hydrocoll..

[55] Ono T., Hori K., Nokubi T. (2004/11/01). Pattern of tongue pressure on hard palate during swallowing. Dysphagia.

[56] Melsen B., Stensgaard K., Pedersen J. (1979). Sucking habits and their influence on swallowing pattern and prevalence of malocclusion. Eur. J. Orthod..

[57] Villa-Chávez C.E., Patiño-Marín N., Loyola-Rodríguez J.P., Zavala-Alonso N.V., Martínez-Castañón G.A., Medina-Solís C.E. (2013/08/01). Predictive values of thermal and electrical dental pulp tests: a clinical study. J. Endo..

[58] Bolla P.K., Rodriguez V.A., Kalhapure R.S., Kolli C.S., Andrews S., Renukuntla J. (2018/08/01). A review on pH and temperature responsive gels and other less explored drug delivery systems. J. Drug Deliv. Sci. Technol..

[59] Cheng S., Butler J.E., Gandevia S.C., Bilston L.E. (2008). Movement of the tongue during normal breathing in awake healthy humans. J. Physiol..

[60] Fregosi R.F., Ludlow C.L. (2014). Activation of upper airway muscles during breathing and swallowing. J. Appl. Physiol..

[61] Nowakowska O., Buliński Z. (2017). Mathematical modelling of heat transport in a section of human forearm. Comp. Assist. Methods Eng. Sci..

[62] Zhu L., Schappeler T., Cordero-Tumangday C., Rosengart A.J. (2009). Advances in Numerical Heat Transfer, vol. 3.

[63] Charkoudian N. (2003). Skin blood flow in adult human thermoregulation: how it works, when it does not, and why. Mayo Clin. Proc..

